# Prevalence and Contributing Factors of Gummy Smile: A Cross‐Sectional Study in Kabul, Afghanistan

**DOI:** 10.1155/bmri/4323534

**Published:** 2026-06-23

**Authors:** Elaha Somaya Ghafary, Sajeya Karimi, Mohammad Hassan Hamrah, Ghulam Sarwar Khalid, Hassina Shadab, Aisha Nawabi, Firoozeh Alipour, Mohammad Eissa Ahmadi, Ahmad Tareq Hamrah

**Affiliations:** ^1^ Department of Periodontics, Kabul University of Medical Science, Kabul, Afghanistan; ^2^ Department of Periodontology, Kabul University of Medical Science, Kabul, Afghanistan; ^3^ Department of Public Health and Health Systems, Nagoya University Graduate School of Medicine, Nagoya, Japan, nagoya-u.ac.jp; ^4^ Department of Pharmacology, Alberoni University, Golbahar, Kapisa, Afghanistan; ^5^ Department of Pediatric Dentistry, Tehran University of Medical Sciences, Tehran, Iran, tums.ac.ir

**Keywords:** excessive gingival display, gummy smile, interlabial distance, prevalence

## Abstract

**Background:**

A gummy smile (GS), or excessive gingival display, is characterized by over 3 mm of gingival exposure when smiling. It affects facial esthetics, often impacting self‐esteem. GS has multifactorial causes, including skeletal, muscular, and dentogingival factors. This study, the first in Afghanistan, examines GS prevalence and associated factors in patients in Kabul.

**Methods and Materials:**

This cross‐sectional study, conducted at the Stomatology Teaching Hospital of Kabul University of Medical Sciences (KUMS) from April to September 2024, included 299 participants aged 10–50 years. Data were collected using structured questionnaires and clinical examinations, with ethical approval from KUMS. Simple random sampling was used, and statistical analyses included chi‐square tests and *t*‐tests to assess associations.

**Results:**

A total of 299 patients were evaluated, with 44% presenting a GS, predominantly females aged 20–30. Significant differences were found in interlabial distance (*p* = 0.001), upper incisor exposure (*p* = 0.001), subnasal to philtrum length (*p* = 0.048), lip hypermobility (*p* = 0.029), and lower/middle third facial ratio (*p* = 0.001).

**Conclusions:**

GS was prevalent among females and younger individuals, particularly those aged 20–30 years, in this Afghan population. Significant factors associated with GS included interlabial distance, upper incisor exposure, lip hypermobility, and subnasal to philtrum length. Further studies with larger, multicenter samples are needed to confirm findings.

## 1. Introduction

A gummy smile (GS), clinically known as excessive gingival display (EGD), is characterized by the exposure of more than 3 mm of gingiva during smiling [1, 2]. While not a pathological condition, GS is often perceived as esthetically unappealing, potentially impacting an individual′s self‐esteem and confidence [3–5]. The balance between teeth, gingiva, and lips is essential for a harmonious smile, and disruptions, such as excessive gingival exposure, can compromise facial esthetics [5, 6].

The prevalence of GS varies globally, with estimates ranging from 10% to 29% in younger populations, particularly among females, due to anatomical differences in lip movement and muscle activity [7–9]. GS is classified by severity: mild (2–4 mm), moderate (4–8 mm), or severe (> 8 mm) [10]. It may present as a continuous anteroposterior gingival band (88%), localized to the anterior (2%) or posterior (6%) regions, or asymmetrically (4%) [11]. Studies report varying prevalence rates, such as 33.8% in Brazilian adolescents [7], 52% in Saudi females [8], and 75.5% in another Saudi cohort [12], highlighting regional differences.

GS has multifactorial causes, including skeletal factors (e.g., vertical maxillary excess and excessive alveolar bone growth), muscular factors (e.g., lip hypermobility and short upper lip), and dentogingival factors (e.g., gingival hyperplasia and altered passive eruption) [13]. The morphofunctional characteristics of the lips, such as length, thickness, and muscle contraction, play a pivotal role in smile esthetics [14]. Treatment for GS is tailored to its etiology and often requires interdisciplinary interventions. Periodontal procedures, such as gingivectomy, esthetic crown lengthening, and lip repositioning surgery, are common. Orthognathic and plastic surgeries, including LeFort I surgery and Botox injections, as well as orthodontic treatments like intrusion of the maxillary anterior segment or entire arch, are also effective options [15].

Despite growing interest in esthetic dentistry and the increasing demand for GS correction, data on its prevalence in specific populations, particularly in developing regions, remain limited. Understanding the frequency and associated factors of GS is crucial for effective treatment planning and improving patient satisfaction. This study, the first in Afghanistan to assess the prevalence and contributing factors of GS, was conducted at a stomatology teaching hospital in Kabul City. By focusing on this condition, the research seeks to provide valuable insights into its occurrence and contribute to a broader understanding of esthetic dental challenges in clinical practice.

## 2. Materials and Methods

This cross‐sectional study was conducted from April to September 2024 at the Stomatology Teaching Hospital, Kabul University of Medical Sciences (KUMS), Afghanistan. A total of 299 participants aged 10–50 years were included. Of these, 25% were dentists, 17.7% were dental students, and the remaining 56.8% were patients attending the hospital. Participants were selected based on global studies reporting higher GS prevalence in younger populations and to encompass a broad age range relevant to esthetic dental concerns [7, 8]. Participants included dentists, dental students, and patients, selected via simple random sampling using a random number generator to minimize selection bias. The source population consisted of individuals present at the hospital during the study period, including dentists, dental students, and patients. These groups were not sampled proportionally; instead, simple random sampling was applied to the combined pool to represent the hospital′s diverse attendees. Ethical approval was obtained from KUMS, and informed consent was collected, with parental consent for participants under 18. The participants comprised dentists, dental students, and patients attending the hospital. They were recruited using random sampling, provided that they voluntarily agreed to participate in the study by answering questionnaires and undergoing clinical examinations to evaluate the prevalence of GS across different age groups and sexes. Consent forms for patients under 18 years old were obtained from their parents. Ethical approval for this study was obtained from the KUMS.

## 3. Inclusion and Exclusion Criteria

The study included participants aged 10–50 years with no harmful habits and exhibiting a facial Pattern 1 (sagittal and vertical balance of the face in frontal and lateral views). Both participants with and without GS were eligible. Exclusion criteria were as follows:

• Individuals are younger than 10 years or older than 50 years.
• Those unable to comprehend or answer the questionnaire due to cognitive impairment, hearing/visual disabilities, or other syndromes.
• Individuals using corticosteroids or other medications that potentially affect gingival conditions.
• Those who declined to participate or withdrew their consent during the study.

Data collection involved 299 individuals who provided informed consent to participate. The data collection process was carried out by four residents from the periodontics department: two female residents for collecting data from women and two male residents for collecting data from men. This ensured gender‐sensitive handling of participants. The study was conducted in the hospital′s outpatient department (OPD) room once a week, with a small number of participants (two adolescents per session) to maintain accuracy and efficiency.

The process included a structured questionnaire designed to gather demographic information (e.g., age and gender) and assess participants′ satisfaction with their smiles. Participants dissatisfied with their smiles were asked to specify what they found unpleasant and why they had not sought treatment. The questionnaire was based on a validated format developed by Makhtar et al. [16], and simple, objective language was used to ensure clarity and comprehension.

To address potential biases, the study implemented measures to mitigate selection, recall, and measurement bias. Selection bias was minimized through the stratified random sampling approach described above. Recall bias was addressed by using a validated questionnaire with clear, objective questions to reduce reliance on subjective memory. Measurement bias was minimized by standardizing clinical examination protocols (detailed below) and training examiners to ensure consistency. Missing data were minimal (< 5%), and cases with missing data were excluded from the relevant analyses for transparency.

## 4. Clinical Examination

Smile evaluations were conducted in the hospital environment under artificial lighting. The examination was performed using sterilized instruments, including a flat mouth mirror, a Williams periodontal probe, and a millimeter stainless steel ruler, which were autoclaved and designated for individual use. Participants were seated in a dental unit with their heads in the natural position, a reproducible and standardized posture in which the head is vertically aligned, and the participant gazes at a distant point at eye level, ensuring a horizontal visual axis [17].

The clinical examination assessed the presence or absence of a GS and recorded variables such as

**Figure 1:**
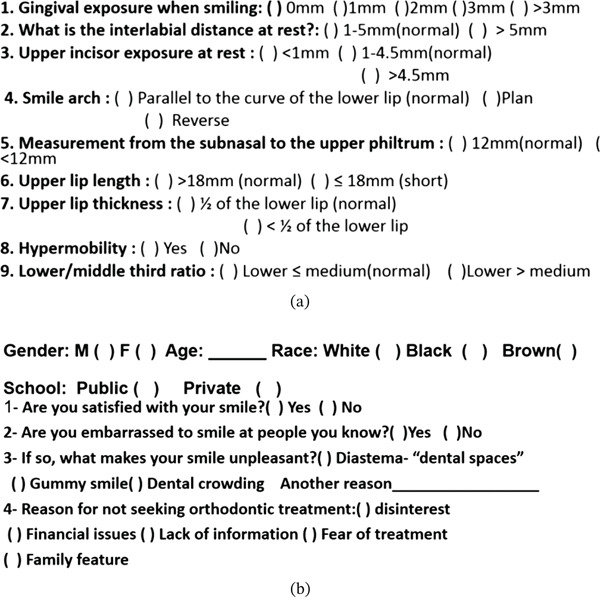
Clinical examination and questionnaire form used to assess gummy smile and related clinical features. (a) Clinical examination form. (b) Participant questionnaire form.

• Interlabial distance at rest.
• Maxillary incisor exposure at rest.
• Smile arc.
• Philtrum length (measured from the base of the nose to the upper lip).
• Upper lip length.
• Lip hypermobility.
• Ratio of the lower/middle third of the face.
• These variables were evaluated following the criteria outlined in a study by Seixas et al. [18] (Figure 1).

## 5. Statistical Analysis

Descriptive statistics were used to summarize demographic and clinical variables. To compare the prevalence of GS across age groups and gender, the Chi‐square test was applied to assess associations between categorical variables (e.g., presence of GS and interlabial distance, upper incisor exposure, smile arch, lip length, philtrum length, and lip hypermobility). All variables were analyzed in categorical form, so only chi‐square tests were used for the reported associations. A *p* value < 0.05 was considered statistically significant. All analyses were conducted using SPSS software, Version 26.0 (IBM Corp., Armonk, New York, United States).

## 6. Results

**Figure 2:**
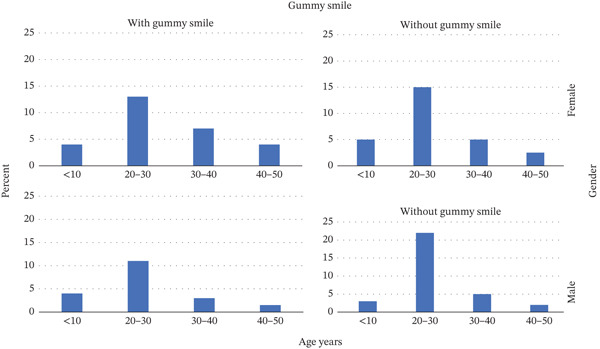
Demographic characteristics of study participants. The figure shows the age and gender distribution of participants with and without a gummy smile (*N* = 299).

A total of 299 patients were evaluated in this study. Among them, 44% presented with a GS, whereas 55.1% did not. The majority of individuals with a GS were females and predominantly in the 20–30 age group (Figure 2).

The interlabial distance at rest was normal (1–5 mm) in 76 participants with a GS and 127 without, whereas 58 with a GS and 38 without had a distance > 5 mm (*p* = 0.001). Upper incisor exposure at rest was less than 1 mm in 16 participants with a GS and 66 without, for a total of 82 individuals. A normal range of 1–4.5 mm was observed in 66 participants with a GS and 93 without (159 total). Exposure greater than 4.5 mm was found in 52 participants with a GS compared to only six without, with a statistically significant difference between the groups (*p* = 0.001). A normal smile arch was observed in 130 participants with a GS and 162 without. Subnasal to philtrum measurements > 12 mm were noted in 79 with a GS and 109 without, with a significant difference (*p* = 0.048). A short upper lip (≤ 18 mm) was found in 125 participants with a GS and 153 without. Hypermobility of the upper lip was observed in 65 participants with a GS and 61 without (*p* = 0.029). The lower/middle third facial ratio was equal in 290 participants, with a significant difference between groups (*p* = 0.001) (Table 1).

**Table 1:** Clinical and demographic characteristics of participants with and without a gummy smile.

Variables		With gummy smile	Without gummy smile	Total	*p* value
Interlabial distance at rest	1–5 mm (normal)	76	127	203	0.001
	> 5 mm	58	38	96	
Upper incisor exposure at rest	< 1 mm	16	66	82	0.001
	1–4.5 mm (normal)	66	93	159	
	> 4.5 mm	52	6	58	
Smile arch	Parallel to the curve of the lip				0.38
	Lower (normal)	130	162	292	
	Plan	4	3	7	
	Reverse				
Measurement from subnasal to upper philtrum	12 mm (normal)	42	51	93	0.048
	< 12 mm	13	5	18	
	> 12 mm	79	109	188	
Upper lip length	> 18 mm (normal)	9	12	21	0.5
	≤ 18 mm (short)	125	153	278	
Hypermobility	Yes	65	61	126	0.029
	No	69	104	173	
Lower/middle third ratio	Yes	125	165	290	0.001
	No	9	0	9	

*Note:* Statistical significance was assessed using Chi‐square tests.

## 7. Discussion

This cross‐sectional study, the first to investigate GS in Afghanistan, found a prevalence of 44% among 299 participants at the Stomatology Teaching Hospital in Kabul, notably higher than global estimates ranging from 10.5% to 29% [9], 33.8% in Brazilian adolescents [7], and 52% in Saudi females [8].

The elevated prevalence in Kabul may be attributed to a combination of genetic, environmental, and socioeconomic factors unique to the Afghan population, including potential craniofacial morphological variations, limited access to esthetic dental interventions, and cultural attitudes toward smile esthetics that may heighten awareness of GS [9, 19]. For instance, the high prevalence aligns closely with Al Sayed et al. [8], who reported 52% in Saudi females, but exceeds Brito et al.′s [7] 33.8% in Brazilian adolescents, suggesting regional differences in GS presentation or detection. These variations could stem from genetic predispositions to vertical maxillary excess or dentoalveolar extrusion prevalent in certain populations, as noted by Jaramillo et al. [9], or from Afghanistan′s limited dental infrastructure, which may result in underdiagnosis and undertreatment of GS, inflating prevalence rates.

The predominance of GS in females (68%) in our study is consistent with prior research indicating that women exhibit greater gingival exposure during smiling due to anatomical differences, such as thinner lips and increased lip elevator muscle activity [7, 19, 20]. Brito et al. [7] reported a 33.8% prevalence in Brazilian adolescents, predominantly females, whereas Al Sayed et al. [8] found 52% in Saudi females, mirroring our findings. This gender disparity may also reflect cultural factors in Afghanistan, where women may place greater emphasis on smile esthetics, leading to higher self‐reporting or clinical detection of GS [16, 21]. The peak prevalence in the 20–30 age group likely results from heightened muscle tone in younger individuals, which enhances upper lip elevation during smiling, as supported by Peck et al. [4] and Jensen et al. [22]. Additionally, younger individuals may be more likely to seek dental consultations for esthetic concerns, potentially contributing to the observed prevalence in this age range [23]. In contrast, older populations may exhibit reduced GS due to age‐related decreases in muscle tone and lip mobility, as noted in prior studies [4, 22, 24].

Significant differences were observed in interlabial distance (> 5 mm, *p* = 0.001), upper incisor exposure (> 4.5 mm, *p* = 0.001), subnasal to philtrum length (*p* = 0.048), and lip hypermobility (*p* = 0.029), corroborating the role of vertical smile dimensions in GS etiology [18]. Seixas et al. [18] identified lip hypermobility as a key etiological factor, which aligns with our finding of significant lip hypermobility in 65 participants with GS compared to 61 without (*p* = 0.029). This suggests that hyperactive lip elevator muscles, such as the levator labii superioris, play a critical role in GS presentation in this population [25]. The significant association of interlabial distance (> 5 mm, *p* = 0.001) contrasts with Peck et al. [4], who suggested that GS is primarily muscular in origin and not directly linked to interlabial distance at rest. This discrepancy may reflect population‐specific skeletal factors, such as a higher prevalence of vertical maxillary excess or dentoalveolar extrusion in Afghan individuals, as supported by Jaramillo et al. [9]. Similarly, the significant association of upper incisor exposure (> 4.5 mm, *p* = 0.001) points to a synergy between dental positioning and muscular dynamics, as excessive incisor exposure may amplify gingival display during smiling [4, 25]. The association with subnasal to philtrum length (> 12 mm, *p* = 0.048) suggests a skeletal contribution, potentially vertical maxillary excess, which contrasts with studies like Pascotto et al. [14] that emphasize muscular causes. This finding may indicate a unique craniofacial profile in the Afghan population, warranting further cephalometric studies to explore skeletal contributions to GS.

This study significantly advances the limited body of GS research in developing regions, where esthetic dentistry data are scarce [9]. The higher prevalence in Kabul compared to high‐income settings, such as Saudi Arabia (52% [8] and 75.5% [12]), may be influenced by socioeconomic barriers, including limited access to periodontal or orthodontic interventions, which could reduce GS visibility in wealthier regions. Cultural perceptions in Afghanistan, where esthetic concerns may be less prioritized due to economic constraints, could also contribute to underreporting or undertreatment, thereby inflating prevalence [16, 21]. Furthermore, the lack of routine dental screening in Afghanistan may result in a higher proportion of undiagnosed GS cases, unlike in high‐income countries with established dental care systems [12]. These factors underscore the need for region‐specific research to inform public health strategies and improve access to esthetic dental care in Afghanistan.

The clinical implications of these findings are significant for Afghan dental practitioners. The high prevalence and identified factors (e.g., lip hypermobility and upper incisor exposure) suggest that treatment planning should prioritize minimally invasive options, such as Botox injections or lip repositioning, for cases driven by muscular factors, and orthodontic or surgical interventions for skeletal‐related GS [15]. The predominance of GS in females may also inform targeted patient education and screening programs, particularly for younger women who may be more concerned about smile esthetics [16]. Additionally, the significant association with subnasal to philtrum length highlights the need for comprehensive craniofacial assessments to tailor treatments effectively [13, 18].

Limitations include the single‐center design, moderate sample size (*n* = 299), and cross‐sectional nature, which preclude causal inferences. However, since data were collected from different groups present in the hospital, the majority of whom were patients, and because these patients belong to diverse age groups, socioeconomic backgrounds, and occupations within the community, hospital‐based sampling may represent a small cross section of the community and can therefore be considered comparable. Additionally, the inclusion of dental professionals and students, who may have greater awareness and concern for dental esthetics, could lead to a higher reported prevalence of GS compared to the general population. Selection bias was mitigated through simple random sampling with a random number generator, and measurement bias was reduced by employing standardized protocols, calibrated instruments, and independent measurements by two examiners. However, the single‐center design limits generalizability to other Afghan populations, and the moderate sample size may not fully capture regional variations. Future studies should employ multicenter, longitudinal designs with larger samples to validate these findings, explore causal pathways, and investigate genetic, environmental, and cultural influences on GS prevalence. Cephalometric analyses could further elucidate skeletal contributions, whereas qualitative studies could explore patient perceptions and barriers to GS treatment in Afghanistan.

## 8. Conclusions

This study, the first in Afghanistan, found a 44% prevalence of GS among 299 participants, predominantly females (68%) aged 20–30 years. Significant differences were observed in interlabial distance (*p* = 0.001), upper incisor exposure (*p* = 0.001), subnasal to philtrum length (*p* = 0.048), lip hypermobility (*p* = 0.029), and lower/middle third facial ratio (*p* = 0.001). These findings highlight the role of muscular and skeletal factors in GS and support targeted treatment strategies, such as lip repositioning or orthodontics, in Afghanistan′s dental practice. Multicenter, longitudinal studies are needed to validate these results and explore regional and genetic influences.

## Funding

No funding was received for this manuscript.

## Conflicts of Interest

The authors declare no conflicts of interest.

## Supporting information


**Supporting Information** Additional supporting information can be found online in the Supporting Information section. The supporting information includes the completed STROBE checklist for cross‐sectional studies (“STROBE‐checklist‐v4‐cross‐sectional_1_.doc”), detailing where each reporting item is addressed within the manuscript.

## Data Availability

The data that support the findings of this study are available on request from the corresponding author. The data are not publicly available due to privacy or ethical restrictions.
