# Supplementary material for: Prevalence and Contributing Factors of Gummy Smile: A Cross‐Sectional Study in Kabul, Afghanistan
Source: Biomed Res Int. 2026 Jun 23;2026:4323534. doi: 10.1155/bmri/4323534 (PMC13291437; doi:10.1155/bmri/4323534)
Supplement: Supplementary file 1 — Supporting Information Additional supporting information can be found online in the Supporting Information section. The supporting information includes the completed STROBE checklist for cross‐sectional studies (“STROBE‐checklist‐v4‐cross‐sectional_1_.doc”), detailing where each reporting item is addressed within the manuscript. [file BMRI-2026-4323534-s001.doc]

STROBE Statement—Checklist of items that should be included in reports of ***cross-sectional studies***

|  | Item No | Recommendation |
| --- | --- | --- |
| **Title and abstract** | 1 | (*a*) Prevalence and Contributing Factors of Gummy Smile: A Cross-Sectional Study in Kabul, Afghanistan |
|  |  | (*b*), **Background:** A gummy smile (GS), or excessive gingival display, is characterized by over 3 mm of gingival exposure when smiling. It affects facial aesthetics, often impacting self-esteem. GS has multifactorial causes, including skeletal, muscular, and dentogingival factors. This study, the first in Afghanistan, examines GS prevalence and associated factors in patients in Kabul.  **Method material**: This cross-sectional study, conducted at the Stomatology Teaching Hospital of Kabul University of Medical Sciences (KUMS) in Kabul, Afghanistan from April to September 2024, included 299 participants aged 15–50 years. Data were collected using structured questionnaires and clinical examinations to evaluate gummy smiles, with ethical approval obtained from KUMS.  **Result:** A total of 299 patients were evaluated, with 44% presenting a gummy smile, predominantly females aged 20–30. Significant differences were found in interlabial distance (p = 0.001), upper incisor exposure (p = 0.001), subnasal to philtrum length (p = 0.048), lip hypermobility (p = 0.029), and lower/middle third facial ratio (p = 0.001).  **Conclusion**: Gummy smile was prevalent among females and younger individuals, particularly those aged 20–30 years, in this Afghan population. Significant factors associated with gummy smile included interlabial distance, upper incisor exposure, lip hypermobility, and subnasal to philtrum length. |
| Introduction | | |
| Background/rationale | 2 | This study is the first research in Afghanistan to assess the prevalence and contributing factors of GS |
| Objectives | 3 | The aim of this study is finding the prevalence of gummy smile |
| Methods | | |
| Study design | 4 | This study is cross-sectional study was conducted between April 2024 and September 2024 at a stomatology teaching hospital in Kabul City, Afghanistan. |
| Setting | 5 | This study was done in stomatology teaching hospital from dentists, dental students and those patient which are come to hospital from April 2024 and September 2024 without follow up data |
| Participants | 6 | The study included participants aged 10–50 years, with no harmful habits, and exhibiting a facial Pattern 1 (sagittal and vertical balance of the face in frontal and lateral views). Both participants with and without gummy smiles were eligible. Exclusion criteria were as follows:  Individuals younger than 10 years or older than 50 years.  Those unable to comprehend or answer the questionnaire due to cognitive impairment, hearing/visual disabilities, or other syndromes.  Individuals using corticosteroids or other medications potentially affecting gingival conditions.  Those who declined to participate or withdrew their consent during the study.  And The participants comprised dentists, dental students, and patients attending the hospital. They were recruited using random sampling, provided they voluntarily agreed to participate in the study by answering questionnaires and undergoing clinical examinations |
| Variables | 7 | Prevalence of gummy smile |
| Data sources/ measurement | 8* | *OPD* ward of Kabul stomatology hospital |
| Bias | 9 | First part of our questioner was self-reported and some of patient with gummy smile were stratified with their smile |
| Study size | 10 | 299 person include patient, dentist dental student |
| Quantitative variables | 11 | A total of 299 patients were evaluated in this study. Among them, 44% presented with a gummy smile, while 55.1% did not. The majority of individuals with a gummy smile were females and predominantly in the 20–30 age group. |
| Statistical methods | 12 | we inserted our data in excel program and after that import data in SPSS version 21 and used descriptive analysis to find prevalence of gummy smile |
|  |  | (*b*) Describe any methods used to examine subgroups and interactions |
|  |  | (*c*) Explain how missing data were addressed |
|  |  | (*d*) If applicable, describe analytical methods taking account of sampling strategy |
|  |  | (*e*) Describe any sensitivity analyses |
| Results | | |
| Participants | 13* | A total of 299 patients were evaluated in this study. Among them, 44% presented with a gummy smile, while 55.1% did not. The majority of individuals with a gummy smile were females and predominantly in the 20–30 age group. |
|  |  | N/A |
|  |  | N/A |
| Descriptive data | 14* | Among them, 44% presented with a gummy smile, while 55.1% did not. The majority of individuals with a gummy smile were females and predominantly in the 20–30 age group. |
|  |  | N/A |
| Outcome data | 15* | The interlabial distance at rest was normal (1–5 mm) in 76 participants with a gummy smile and 127 without, while 58 with a gummy smile and 38 without had a distance >5 mm (p = 0.001). Upper incisor exposure at rest was less than 1 mm in 16 participants with a gummy smile and 66 without, for a total of 82 individuals. A normal range of 1–4.5 mm was observed in 66 participants with a gummy smile and 93 without (159 total). Exposure greater than 4.5 mm was found in 52 participants with a gummy smile compared to only 6 without, with a statistically significant difference between the groups (p = 0.001). A normal smile arch was observed in 130 participants with a gummy smile and 162 without. Subnasal to philtrum measurements >12 mm were noted in 79 with a gummy smile and 109 without, with a significant difference (p = 0.048). A short upper lip (≤18 mm) was found in 125 participants with a gummy smile and 153 without. Hypermobility of the upper lip was observed in 65 participants with a gummy smile and 61 without (p = 0.029). The lower/middle third facial ratio was equal in 290 participants, with a significant difference between groups (p = 0.001). |
| Main results | 16 | a prevalence of 44% was observed in individuals with a higher prevalence among females aged 20–30 years |
|  |  |  |
|  |  |  |
| Other analyses | 17 | N/A |
| Discussion | | |
| Key results | 18 | A total of 299 patients were evaluated in this study. Among them, 44% presented with a gummy smile, while 55.1% did not. The majority of individuals with a gummy smile were females and predominantly in the 20–30 age group |
| Limitations | 19 | Our limitations include the single-center design, small sample size, and cross-sectional methodology, which preclude causal inferences |
| Interpretation | 20 | N/A |
| Generalisability | 21 | N/A |
| Other information | | |
| Funding | 22 | N/A |

*Give information separately for exposed and unexposed groups.

**Note:** An Explanation and Elaboration article discusses each checklist item and gives methodological background and published examples of transparent reporting. The STROBE checklist is best used in conjunction with this article (freely available on the Web sites of PLoS Medicine at http://www.plosmedicine.org/, Annals of Internal Medicine at http://www.annals.org/, and Epidemiology at http://www.epidem.com/). Information on the STROBE Initiative is available at www.strobe-statement.org.
